# Assessment of level-walking aperiodicity

**DOI:** 10.1186/1743-0003-3-28

**Published:** 2006-12-07

**Authors:** Fabrizio Pecoraro, Claudia Mazzà, Mounir Zok, Aurelio Cappozzo

**Affiliations:** 1 Department of Human Movement and Sport Sciences, Istituto Universitario di Scienze Motorie, Rome, Italy

## Abstract

**Background:**

In gait analysis, walking is assumed to be periodic for the sake of simplicity, despite the fact that, strictly speaking, it can only approximate periodicity and, as such, may be referred to as pseudo-periodic. This study aims at: 1) quantifying gait pseudo-periodicity using information concerning a single stride; 2) investigating the effects of walking pathway length on gait periodicity; 3) investigating separately the periodicity of the upper and lower body parts movement; 4) verifying the validity of foot-floor contact events as markers of the gait cycle period.

**Methods:**

Ten young healthy subjects (6 males, 23 ± 5 years) were asked to perform various gait trials, first along a 20-m pathway that allowed reaching a steady-state condition, and then along an 8-m pathway. A stereophotogrammetric system was used to reconstruct the 3D position of reflective markers distributed over the subjects' body. Foot contact was detected using an instrumented mat. Three marker clusters were used to represent the movement of the whole body, the upper body (without upper limbs), and the lower body, respectively. Linear and rotational kinetic, and gravitational and elastic potential "energy-like" quantities were used to calculate an index J(t) that described the instantaneous "mechanical state" of the analysed body portion. The variations of J(t) in time allowed for the determination of the walking pseudo-period and for the assessment of gait aperiodicity.

**Results:**

The suitability of the proposed approach was demonstrated, and it was shown that, for young, healthy adults, a threshold of physiological pseudo-periodicity of walking at natural speed could be set. Higher pseudo-periodicity values were found for the shorter pathway only for the upper body. Irrespective of pathway length, the upper body had a larger divergency from periodicity than the lower body. The error that can be made in estimating the gait cycle duration for the upper body from the heel contacts was shown to be significant.

**Conclusion:**

The proposed method can be easily implemented in gait laboratories to verify the consistency of a recorded stride with the hypothesis of periodicity.

## Background

When performing gait analysis, subjects are normally asked to walk at a constant speed of progression (at steady-state). The resulting estimated kinematic and kinetic quantities are assumed to be periodic, and, as such, are described with reference to a single walking cycle [1,2]. This cycle is commonly defined by the interval of time (T) that starts at the initial contact of one foot and ends at the following contact of the same foot [2,3]. It is evident that the biological phenomenon that is dealt with is assumed to be periodic for the sake of simplicity, but, strictly speaking, it can only approximate periodicity and it may be referred to as pseudo-periodic [4].

The cyclic nature of gait data patterns emerges from gait initiation and is ultimately clearly identifiable only during steady-state pace. Since such pace is reached after negotiating some steps [5], short walking pathways, as found in many gait laboratories, might be one of the causes that contribute to the pseudo-periodicity in the recorded data. This might in turn reflect into an undesired augmented variability in the data, hiding the valuable information obtainable from variability analysis [6-8].

When talking about gait periodicity, reference should be made to the mechanical state of the locomotor system and to its reiteration after a given interval of time. Relevant state variables can be derived from the quantities normally measured in the gait analysis laboratory, such as joint angles [9] or mechanical energies [5]. Starting from a reference instant of time, the system mechanical state variation, determined in any subsequent instant of time, provides a measure of the aperiodicity of the phenomenon as observed in that interval of time. When this variation reaches a minimum value, aperiodicity is at a minimum and, therefore, the corresponding interval of time may be considered as the best estimate of the pseudo-period ($\hat{\text{T}}$) and the relevant mechanical state variation as a measure of pseudo-periodicity or aperiodicity. The normally used foot-floor contacts represent a very partial description of the locomotor system mechanical state and, as such, may be not fully adequate for the determination of the walking pseudo-period.

The above considerations lead to the formulation of the following questions, which this paper aims at providing an answer:

1. Given a gait stride, perceived by the walking subject as performed at steady-state, how far is it from being a cycle of a periodic phenomenon and is it associated with a pseudo-periodic or an aperiodic gait?

2. Does a limited walking pathway length cause an increase in gait pseudo-periodicity?

3. As far as the above listed issues are concerned, is there a difference between the pseudo-periodic characteristics of the movements of the lower part and those of the upper part of the body?

4. How valid is the foot-floor contact method for determining the duration (pseudo-period) of the walking cycle?

In principle, the hypothesis of periodicity should be verified and quantitatively assessed by comparing relevant quantities recorded during a series of consecutive strides. However, this is hardly ever possible when using stereophotogrammetry and dynamometry, since the measurement volume normally does not host more than three consecutive steps. A method for the quantification of the discrepancy between periodicity and pseudo-periodicity or aperiodicity of walking through the observation of a single gait stride will be proposed in this paper. The information provided by this method is expected to be useful for two reasons: from a heuristic point of view, it allows an insight into a possible methodological, external, cause of the variability of gait strides [10], and, from a practical standpoint, it allows for a control of the consistency of the observed gait stride with the hypothesis of steady state.

## Materials and methods

### Subjects and protocol

Ten young healthy subjects (6 males, 23 ± 5 years, 62 ± 12 kg, 1.68 ± 0.08 m) volunteered for the study and signed an informed written consent. Subjects participated in two sets of experiments each characterised by a different walking pathway length. They were asked to walk along the linear pathways at three different self-selected speeds of progression: slow (SS, "walk at a slow speed"), natural (NS, "walk naturally") and fast (FS, "walk as fast as you can"). In all cases the subjects were explicitly asked to reach and maintain a constant speed of progression. Three trials were performed for each condition.

### Instrumentation

A purposely built instrumented mat was used to measure the beginning (t_b_) and end (t_e_) of a stride determined by the consecutive contacts of the same foot with the mat, and relevant stride duration (T) was then computed as T = t_e _– t_b_. Adhesive 5 mm wide copper stripes were attached parallel to each other at a 3 mm distance along a 4 m length linoleum mat. Alternative stripes were connected to an electric circuit so that, when short circuited, a signal was generated. Two independent circuits were constructed for right and left foot. Subjects wore custom designed socks, the bottom part of which was covered with conductive material. The accuracy of the mat was assessed by comparing its data to those simultaneously acquired with a strain gauge force plate (Bertec Corporation, Ohio, USA, sample frequency = 120 samples/s) while a subject stepped on it. The first sample at which the vertical force was greater than its mean value plus two standard deviations recorded for 1 s while the force plate was unloaded, was chosen as indicator of the foot contact. The differences found between the time events detected with the mat and with the force plate were computed for ten different trials, and were always lower than 0.025 s.

A nine-camera VICON^® ^system (Oxford Metrics, Oxford, UK) was used to reconstruct the 3D positions, relative to the stereophotogrammetric set of axes, of 19 markers placed on the body of the subjects. The markers were placed on the head (three markers attached on an elastic band), trunk (spinal process of the seventh cervical vertebra, acromion processes), pelvis (anterior superior iliac spines, midpoint between the posterior superior iliac spines), and lower limbs (greater trochanters, femoral lateral epicondyles, lateral malleoli, calcanei, and second metatarsal heads). From now on, the cluster composed by all the above listed markers will be referred to as whole body (WB) cluster. Two sub-clusters will also be considered: the lower body (LB) cluster, including the 13 markers located on pelvis and lower limbs, and the upper body (UB) cluster, including the 9 markers located on head, trunk and pelvis. While defining the latter cluster, it was decided not to include upper limb markers because of the low sensitivity of the overall gait pattern to the movement of the upper limbs, which, for this reason, may tend to be more aperiodic than that of the rest of the body. In addition, most gait analysis protocols do not include these segments.

Stereophotogrammetric and mat data were simultaneously collected at a sampling frequency of 120 samples/s.

### Experiments

As mentioned previously, two sets of experiments were performed. The first set aimed at placing the subjects in the best condition for reaching steady-state walking and at properly assessing periodicity by observing more than a single stride. The second set aimed at simulating a standard laboratory situation where only a single stride per limb fits in the measurement volume and the walking pathway length is limited.

The first set of experiments was performed exploiting the entire length of a 20 × 8 m laboratory such that subjects were able to walk for at least twelve consecutive strides and the stereophotogrammetry measurement volume hosted two strides per limb (among the fifth, sixth and seventh stride). This pathway allowed the subjects to reach what they perceived to be a steady-state walking pace [5].

The second set of experiments used the same protocol as described above, but was carried out along an 8-m pathway and within a stereophotogrammetric measurement volume that hosted only three consecutive steps.

### Data analysis

Through a rigid transformation, 3-D marker position data were represented relative to a laboratory set of axes, the X axis of which was aligned with the analysed subject mean speed of progression, and the Y axis was vertical. This data was filtered through a low-pass fourth-order Butterworth filter with a cut off frequency of 8 Hz [11] and was used to describe the variations of the mechanical state of the subjects' whole body, and of its upper and lower parts.

Each cluster was considered as an ensemble of particles with equal mass and was represented, in each sampled instant of time during movement and relative to the laboratory frame, by the global position vector (^g^**p**) of its centre of mass and by the orientation matrix (^g^**R**) of an arbitrarily chosen set of local axes. To this purpose, the singular value decomposition technique was used [12]. The position vectors of the markers in the local frame is referred to as ^l^**p**. Using this information, energy-like quantities were calculated and used to describe the instantaneous "mechanical state" variation of each cluster and, in turn, of each related body system. Such variations were calculated relative to the reference instant of time t_b_.

The vertical coordinate *h*(t) of the marker cluster centre of mass was considered to represent a gravitational potential energy-like quantity G(t). Its variation was calculated as:

ΔG(t) = *h*(t) - *h*(t_b_).     (1)

The first derivative of the centre of mass position vector was estimated via a three-point central difference differentiation method. The modulus of the instantaneous velocity thus obtained (*v*(t)) was used to calculate a linear kinetic energy-like quantity K(t). Its variation was given by:

ΔK(t) = *v*^2 ^(t) - *v*^2 ^(t_b_). (2)

The instantaneous angular velocity (*ω*(t)) of the cluster was computed from the orientation matrix ^g^**R **[13]. The modulus of *ω*(t) was used to calculate a rotational kinetic energy-like quantity R(t). Its variation was given by:

ΔR(t) = *ω*^2 ^(t) - *ω*^2 ^(t_b_).     (3)

Besides height and velocities variations, during movement the clusters may undergo a variation in orientation and a deformation, both of which were described by elastic potential energy-like quantities. The orientation variation of a cluster between time t_b _and time t may be thought to correspond to a rotation of the local set of axes about the corresponding finite helical axis against an elastic torsional constraint. From the orientation matrices of the cluster at times t_b _and t, the relevant rotation vector (**θ**(t)) was calculated [14]. The following torsional elastic potential energy-like quantity was, thus, determined:

ΔT(t) = ||**θ**(t)||^2^.     (4)

Similarly, the variation of the markers local position vectors between time t_b _and time t allowed for the calculation of another elastic potential energy-like quantity associated with marker cluster deformation:

$\Delta \text{D}(\text{t})=\frac{\sum_{\text{i}=1}^{\text{N}}{\left\|{\;}^{\text{l}}p_{\text{i}}(\text{t})-{\;}^{\text{l}}p_{\text{i}}({\text{t}}_{\text{b}})\right\|}^{2}}{\text{N}},\;\left(5\right)$

where N is the number of markers of the relevant cluster.

A measure of the system mechanical state variation, in any observed interval of time, could be obtained through the sum of the absolute values of the above-defined energy-like quantities. However, since such quantities have arbitrary dimensions, their values are incomparable and should hence be normalised. The maximum amplitude of one (arbitrarily chosen) of the energy-like quantities could be considered as a reference normalisation factor for the others. In such way, the variation of the mechanical state of the system can be calculated according to the following weighed sum:

$\Delta \text{E}(\text{t})=\frac{\left|\Delta \text{G}(\text{t})\right|}{{\text{k}}_{\text{G}}}+\frac{\left|\Delta \text{K}(\text{t})\right|}{{\text{k}}_{\text{K}}}+\frac{\left|\Delta \text{R}(\text{t})\right|}{{\text{k}}_{\text{R}}}+\frac{\left|\Delta \text{T}(\text{t})\right|}{{\text{k}}_{\text{T}}}+\frac{\left|\Delta \text{D}(\text{t})\right|}{{\text{k}}_{\text{D}}},\;\left(6\right)$

where k_G_, k_K_, k_R_, k_T_, and k_D _are weighing constants. These constants, for the *i*-th trial, are arbitrarily calculated considering (for example) the maximum amplitude of gravitational potential energy-like quantity as the reference normalisation factor (i.e. setting k_G _= 1):

${\text{k}}_{{\text{G}}_{\text{i}}}=\frac{\max\left|\Delta {\text{G}}_{\text{i}}(\text{t})\right|}{\max\left|\Delta {\text{G}}_{\text{i}}(\text{t})\right|}=1\;\left(7\text{a}\right)$

${\text{k}}_{{\text{K}}_{\text{i}}}=\frac{\max\left|\Delta {\text{K}}_{\text{i}}(\text{t})\right|}{\max\left|\Delta {\text{G}}_{\text{i}}(\text{t})\right|}\;\left(7\text{b}\right)$

${\text{k}}_{{\text{R}}_{\text{i}}}=\frac{\max\left|\Delta {\text{R}}_{\text{i}}(\text{t})\right|}{\max\left|\Delta {\text{G}}_{\text{i}}(\text{t})\right|}\;\left(7\text{c}\right)$

${\text{k}}_{{\text{T}}_{\text{i}}}=\frac{\max\left|\Delta {\text{T}}_{\text{i}}(\text{t})\right|}{\max\left|\Delta {\text{G}}_{\text{i}}(\text{t})\right|}\;\left(7\text{d}\right)$

${\text{k}}_{{\text{D}}_{\text{i}}}=\frac{\max\left|\Delta {\text{D}}_{\text{i}}(\text{t})\right|}{\max\left|\Delta {\text{G}}_{\text{i}}(\text{t})\right|}\;\left(7\text{e}\right)$

However, if different trials are to be compared, a fixed reference value of the constants should be chosen for all of them. Since no reference values were available to this purpose, previously (unpublished) available kinematic gait data, recorded at natural speed from a similarly aged group of 15 healthy subjects adopting the same instrumentation and marker set as the ones in the present study, were used. The values of the constants were computed as in (7a-7e) for each trial, and their mean values (k_G _= 1.00, k_K _= 0.04, k_R _= 0.27, k_T _= 0.05, k_D _= 0.70) were used in the rest of the study.

The variation of the state of the system during a gait cycle, starting from a foot contact (t_b_) and normalised with respect to its maximum value, was assessed by means of the index:

$\text{J}(\text{t})=\frac{\Delta \text{E}(\text{t})}{\max\Delta \text{E}(\text{t})}\times 100.\;\left(8\right)$

The sought aperiodicity index was computed as:

*J*_*min *_= min (J(t)).     (9)

The larger the *J*_*min *_value, the further the analysed gait is from a periodic process. The time instant for which J(t) = *J*_*min *_is proposed as an estimate of the end of the period ($\hat{\text{ t}}$_e_), which can be used to determine the pseudo-period $\hat{\text{T}}$ = $\hat{\text{t}}$_e _- t_b_. The value assumed by J(t) at t_e_, i.e. that measured by the mat at the end of the stride, will be referred to as *J*_e_.

To assess the sensitivity of the index *J*_*min *_to the values of the constants, a set of 100 different combinations of values was generated by randomly varying them in the ranges defined by their corresponding mean values plus or minus one standard deviation, computed over the above described 75 trials (k_G _= 1.00, k_K _= 0.04 ± 0.03, k_R _= 0.27 ± 0.15, k_T _= 0.05 ± 0.03, k_D _= 0.70 ± 0.19).

As previously mentioned, gait aperiodicity depends on step length, cadence, and width, which can differently affect cluster kinematics: step length variation can be expected to mostly affect *h*(t), and partly *v*(t) and ^l^**p**(t); step cadence variation mostly affects *v*(t); step width variation mostly affects *h*(t) and ^l^**p**(t). Thus, it can be hypothesised that within the same stride, the quantities ΔG(t) and ΔD(t) are the most sensitive to changes in step length and width, and the index ΔK(t) to changes in step length and cadence. Moreover, the two terms ΔR(t) and ΔT(t) are expected to be negligible when walking straight. In such case, the equations (6), (8) and (9) can be replaced by the following:

$\Delta \hat{\text{E}}(\text{t})=\frac{\left|\Delta \text{G}(\text{t})\right|}{{\text{k}}_{\text{G}}}+\frac{\left|\Delta \text{K}(\text{t})\right|}{{\text{k}}_{\text{K}}}+\frac{\left|\Delta \text{D}(\text{t})\right|}{{\text{k}}_{\text{D}}},\;\left(6\text{a}\right)$

$\hat{\text{J}}(\text{t})=\frac{\Delta \hat{\text{E}}(\text{t})}{\max\Delta \hat{\text{E}}(\text{t})}\times 100,\;\left(8\text{a}\right)$

$\hat{J}$_*min *_= min ($\hat{\text{J}}$(t)). (9a)

The above described hypotheses were verified by means of *ad-hoc *constrained tests. One subject (male, 23 years, 1.70 m, 70 kg) was asked to follow the auditory input of a metronome to modulate step cadence (C), and the visual input of markers on the floor to control step length (L) and width (W) while walking along the 8-m pathway. L, C, and W were first kept unconstrained, and then made to vary, one at a time, from step to step (ΔL = 0.4 m, ΔC = 1 step/s, ΔW = 0.2 m).

### Statistical analysis

The coefficient of determination (R^2^) was used, for both mat-measured period (T) and pseudo-period ($\hat{\text{T}}$), to assess the equivalence of the duration of the first and second stride of the same trial. A two-way ANOVA analysis was used to assess the effects of two between group factors: speed (three levels: SS, NS, and FS) and pathway length (two levels: short, SP, and long, LP). When significant differences (p < 0.05) were found, a post-hoc analysis was performed using an unpaired samples two-tailed t-test with Bonferroni correction (significance level: p = 0.017). Finally, a two-tailed t-test for paired samples (p = 0.05) was used to compare the results obtained for the three clusters of markers and to assess the differences between T and $\hat{\text{T}}$.

## Results

The first three steps of the analysis consisted in the validation of the proposed method in terms of: robustness of the index *J*_*min *_to the variation of the constants k; sensitivity of the energy-like indices to the gait characteristics; suitability of the method to detect periodicity by observing changes between subsequent strides.

When varying the five constants in the computed ranges, the index *J*_*min *_varied by less than 10% of its initial value and the corresponding $\hat{\text{T}}$ remained unaltered.

**Figure 1:**
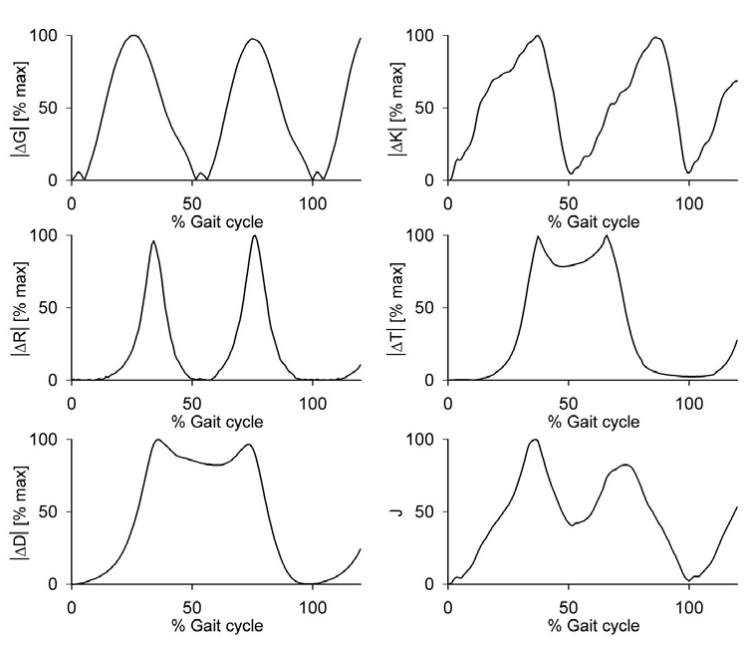
Example of the time patterns of the "energy-like" indices used to describe the mechanical state of the system during one gait trial. The data along the abscissa are normalised with respect to the duration of the first stride, determined as $\hat{\text{T}}$ = $\hat{\text{t}}$_e _- t_b_.

Fig. 1 illustrates, for a representative LP trial, an example of the variations of the WB indices ΔG(t), ΔK(t), ΔR(t), ΔT(t), ΔD(t), and J(t) from their values at t_b_. The sensitivity of these indices to stride parameter variations is illustrated by the data reported in Table 1: as expected, *J*_*min *_was sensitive to variations in step length, cadence, and width. When length and cadence varied, the contribution, at instant $\hat{\text{t}}$_e_, of ΔG and ΔK to the overall *J*_*min *_reached 71% and 61%, respectively.

**Table 1:** Results of the trials performed by one subject in controlled experimental conditions (the gait factors, namely step length, cadence, and width, varied from step to step: ΔL = 0.4 m, ΔC = 1step/s, ΔW = 0.2 m, respectively).

Gait Factor	*J*_*min *_(%)	ΔG (%*J*_*min*_)	ΔK (%*J*_*min*_)	ΔR (%*J*_*min*_)	ΔT (%*J*_*min*_)	ΔD (%*J*_*min*_)
None	10	81	10	1	5	3
Length	29	71	5	3	3	19
Cadence	21	13	61	1	5	20
Width	37	46	36	1	4	13

The percentage contributions of the variation of the gravitational potential (ΔG), linear (ΔK) and rotational (ΔR) kinetic, torsional (ΔT) and deformation (ΔD) elastic potential energy-like quantities to the total value of Jmin, as computed at $\hat{\text{ t}}$, are shown.

The results in Table 1 show that, as hypothesised, the two terms ΔR(t) and ΔT(t) can be neglected when walking straight. These indices, in fact, contributed to the overall *J*_*min *_by no more than 5%. As a result, from now onward, the index $\hat{J}$_*min *_will be used for the assessment of periodicity.

**Figure 2:**
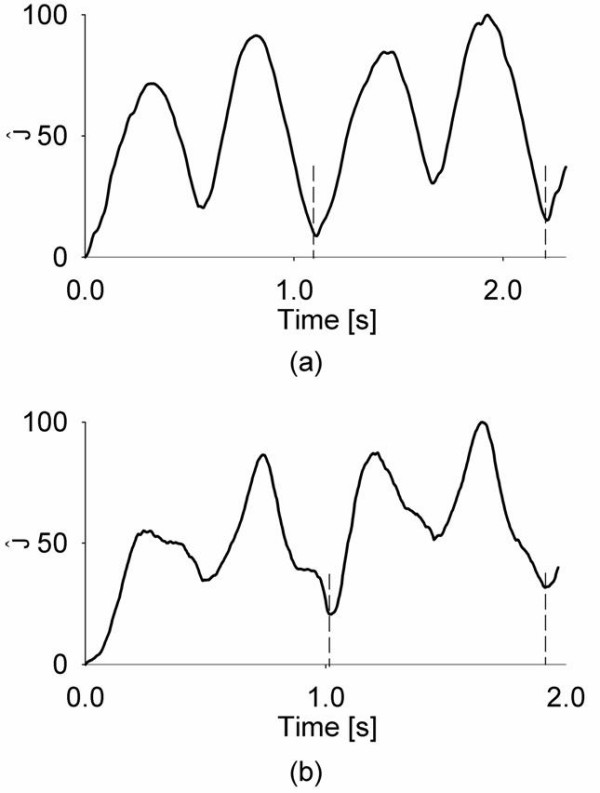
The time patterns of $\hat{\text{J}}$(t) are reported for two different trials, one at steady-state (a) and one during which the subject was deliberately accelerating (b). The vertical dashed lines indicate the t_e _values recorded from the mat.

An example of the $\hat{\text{J}}$(t) time patterns obtained for the WB cluster between subsequent strides is reported in Fig. 2. In particular, the data obtained during the constrained tests of a representative subject when asked to walk at steady-state (Fig. 2a) and when asked to vary progression speed freely between the two strides (Fig. 2b) is illustrated. In the reported figure, during steady-state, the $\hat{J}$_*min *_values were similar in the two subsequent strides (8.7% and 7.8%, respectively, computed within the relevant T) and were lower than those obtained when the subject was accelerating during the first stride (32.2% and 10.3%, respectively).

Due to the longer stride length at the faster speed, it was not possible to collect reliable data for two consecutive strides in the FS experiments performed by the subjects along the LP. For this reason, only the SS and NS trials were included in this first part of the analysis. These trials actually resulted to be pseudo-periodic, since:

a) consecutive strides had the same duration, as shown by the high coefficient of determination between the duration of the first (T_1_) and of the second (T_2_) stride (R^2 ^= 0.97 for T_1 _and T_2 _and R^2 ^= 0.90 for $\hat{\text{T}}$_1 _and $\hat{\text{T}}$_2_), and

b) the two time curves of $\hat{\text{J}}$(t) obtained in T_1 _and T_2 _(and resampled to 100 samples) were highly correlated (Pearson correlation coefficient *r *= 0.92 ± 0.13) and very similar to each other (RMS = 9 ± 8%). The same stands for the curves computed using $\hat{\text{T}}$_1 _and $\hat{\text{T}}$_2 _(*r *= 0.92 ± 0.12 and RMS = 10 ± 7%).

The values of $\hat{J}$_*min *_obtained for this sub-set of experiments (WB: 9 ± 9%; LB: 7 ± 6%; UB: 16 ± 15%) can be used to set a threshold between pseudo-periodicity and aperiodicity for the three clusters of markers.

All the 90 LP trials were then compared with the SP trials for the three speeds of execution of the task. Mean speeds of progression, calculated as the product between stride length and stride frequency, did not significantly change between SP and LP trials (SS: 0.93 ± 0.22 ms^-1 ^vs 0.84 ± 0.16 ms^-1^, NS: 1.16 ± 0.18 ms^-1 ^vs 1.20 ± 0.25 ms^-1^, and FS: 2.21 ± 0.14 ms^-1 ^vs 1.91 ± 0.52 ms^-1^).

As reported in Table 2, the results of the ANOVA showed that the two factors, speed and pathway length, when considered separately, affected $\hat{J}$_*min *_of the UB cluster only: relevant $\hat{J}$_*min *_values increased with increasing speed and shortening of the pathway length (Table 3).

**Table 2:** Results of the ANOVA performed on the $\hat{J}$_*min *_values obtained for the three clusters of markers.

Factor	$\hat{J}$**_*min *_- WB**		$\hat{J}$**_*min *_- LB**		$\hat{J}$**_*min *_- UB**	
	F	p	F	p	F	p
Speed	3.262	0.062	2.526	0.108	7.795	0.004
Pathway Length	2.457	0.151	1.432	0.262	10.862	0.009
Speed × Pathway Length	2.914	0.080	1.192	0.327	2.684	0.095

**Table 3:** Mean values (standard deviation) obtained for the two sets of experiments (long pathway, LP, and short pathway, SP) in the different trial types: slow (SS), natural (NS) and fast (FS) walking speeds.

Trial type	$\hat{J}$**_*min *_- WB (%)**		$\hat{J}$**_*min *_- LB (%)**		$\hat{J}$**_*min *_- UB (%)**	
	LP	SP	LP	SP	LP	SP
SS	9 (7)	11 (2)	7 (5)	8 (2)	14 (10)*^	20 (5)*^
NS	9 (6)	12 (2)	7 (5)	9 (1)	10 (8)^	20 (5)^§^*^
FS	10 (8)	15 (4)	8 (6)	10 (3)	15 (11)°*^	27 (7)°^§^*^

The values of the index $\hat{J}$ are reported for the whole body (WB), lower body (LB) and upper body (UB) marker clusters. (°significant differences between FS and NS; § significant differences between LP and SP; *significant differences between UB and WB; ^significant differences between UB and LB).

The results of the comparison among the three body clusters are highlighted in Table 3, where the mean (standard deviation) values of $\hat{J}$_*min *_computed in all the experimental conditions are shown. Whereas no significant differences were found between the WB and LB, the UB almost always showed the highest $\hat{J}$_*min *_values. The only exception was relative to walking at NS along LP in which case $\hat{J}$_*min *_was not significantly different between WB and UB.

Table 4 shows the results of the comparison between the stride durations, once estimated ($\hat{\text{T}}$) using the marker clusters and once measured with the mat (T) using the heel strike. The differences between the two quantities were, on average, less than or equal to 3% of T for WB and LB and less than or equal to 7% of T for UB. Significant differences were observed for all clusters at both SS and NS, except for WB and LB when walking along LP.

**Table 4:** Mean values (standard deviation) of the differences between the stride duration values measured with the mat (T) and those estimated with the index $\hat{J}$_*min *_($\hat{\text{T}}$), expressed as a percentage of T.$\hat{\text{T}}$

Trial type	WB		LB		UB	
	LP	SP	LP	SP	LP	SP
SS	1 (1)	**2 (1)**	1 (1)	**1 (1)**	**3 (1)**	**3 (1)**
NS	2 (1)	**2 (1)**	1 (1)	**2 (1)**	**4 (1)**	**3 (1)**
FS	2 (1)	3 (2)	2 (1)	3 (2)	7 (9)	4 (5)

Results are reported for the two sets of experiments (long pathway, LP, and short pathway, SP). The values in bold indicate the experimental conditions in which the differences between T and $\hat{\text{T}}$ were significant.

Table 5 shows the results of the ANOVA performed on the $\hat{J}$_e _values: for all clusters, only the speed factor caused a significant change in $\hat{J}$_e_, with the highest values found at FS along SP (Table 6); for the UB cluster, $\hat{J}$_e _was not significantly different at FS and SS.

**Table 5:** Results of the ANOVA performed on the $\hat{J}$_*e *_values obtained for the three clusters of markers.

Factor	$\hat{J}$**_*e *_- WB**		$\hat{J}$**_*e *_- LB**		$\hat{J}$**_*e *_- UB**	
	F	p	F	p	F	p
Speed	14.624	0.000	21.453	0.000	11.086	0.001
Pathway Length	1.947	0.196	1.002	0.343	3.462	0.096
Speed × Pathway Length	0.801	0.464	0.489	0.621	0.394	0.680

**Table 6:** Mean values (standard deviation) obtained for the two sets of experiments (long pathway, LP and short pathway, SP) in the different trial types: slow (SS), natural (NS) and fast (FS) walking speeds.

Trial type	$\hat{J}$**_*e *_- WB (%)**		$\hat{J}$**_*e *_- LB (%)**		$\hat{J}$**_*e *_- UB (%)**	
	LP	SP	LP	SP	LP	SP
SS	11 (7)	13 (3)	9 (5)	10 (2)	20 (13)*^	25 (5)*^
NS	11 (6)	15 (3)	8 (4)	11 (2)	16 (9)*^	23 (5)*^
FS	14 (11)	21 (6)^†^°	13 (10)	16 (4)^†^°	23 (14)*^	35 (9)°*^

The values of the index $\hat{J}$ are reported for the whole body (WB), lower body (LB) and upper body (UB) marker clusters. († significant differences between FS and SS; °significant differences between FS and NS; *significant differences between UB and WB; ^significant differences between UB and LB).

The use of t_e _instead of $\hat{\text{t}}$_e _led to an increase in the estimate of gait pseudo-periodicity: the values of $\hat{\text{J}}$(t) at t_e _($\hat{J}$_*e*_, Table 6) were significantly higher than those at $\hat{\text{t}}$_e _($\hat{J}$_*min*_, Table 3) for all clusters in all experimental conditions.

## Discussion

The objectives of this study were: 1) to gather information concerning the periodicity of walking cycles and to set a threshold between pseudo-periodic and aperiodic walking; 2) to describe the effects of a limited walking pathway on gait pseudo-periodicity; 3) to assess differences in the movements of the lower and of the upper part of the body; 4) to assess the validity of the foot-floor contact method for determining the duration (pseudo-period) of the walking cycle.

To achieve the above listed objectives, a mechanical energy-like index computed from the kinematic data recorded during one stride only has been devised. This method was validated performing *ad-hoc *experiments which allowed for the comparison of two consecutive strides recorded in a pathway which certainly allowed the subjects to reach the steady-state condition. The results indicated that the proposed index is suitable for the measure of gait aperiodicity using one stride only.

The first two objectives were reached by assessing gait pseudo-periodicity. It was shown that, if considering the whole body cluster, a value of 18% (mean + one standard deviation) of the global variation of the mechanical energy-like index can be considered as a threshold of physiological pseudo-periodicity of young, healthy adult gait. Values below this threshold, in fact, were found when subjects were asked to walk along the 20-m pathway at NS and SS. Gait periodicity seemed reduced when subjects were asked to walk along the 8-m pathway and this was most evident at their maximal speed. These differences, however, were significant only for the upper part of the body.

The third objective of this study required the assessment of the periodicity of the different parts of the human body. In almost all the experimental conditions, the upper part of the body showed higher aperiodicity than the lower part. This behaviour can be explained by the lower number of functional constraints that trunk and head movements have to comply with during gait. Lower limbs, in fact, are responsible for forward progression and must hence act in a quite regular and constrained fashion, whereas head and trunk can, theoretically, freely behave while being "carried" by the lower part of the body [15].

Finally, the fourth objective of the paper was tackled and the validity of considering the foot-floor contact events as markers of the period of gait cycles was assessed. The error (mean + one standard deviation) that can be made in estimating the gait cycle duration for the whole body from the heel contacts, is, on average, less than 3% of the period in long pathway condition at all gait speeds. This error can increase up to 5% while walking at fast speed along the short pathway, and can lead to an increase of the pseudo-periodicity value from 19% to 27%. The different behaviour of lower and upper body described in the above paragraph was confirmed by the differences found between the periods estimated for the two clusters along the 20-m pathway: whereas the period estimated for the lower body was the same as that measured from the foot-floor contacts, noticeable differences were recorded for the upper part of the body. This proves that special attention should be dedicated when the foot-floor contact method is used for detecting the period of the whole and lower body along short pathways, and it should never be considered valid for the upper body.

## Conclusion

This study showed that young, healthy adult human gait is pseudo-periodic, and this is more marked for the upper part of the body. A control of aperiodicity should always be performed if trials are conducted in common gait laboratories. If any instrument is available for the detection of the beginning of a stride, for example a force plate, then the proposed index could be used to accurately estimate stride duration.
